# Non-Operative Considerations in Relation to Parastomal Hernia

**DOI:** 10.3389/jaws.2025.15473

**Published:** 2025-10-15

**Authors:** Z. Malaibari, M. W. Christoffersen, M. Krogsgaard, N. A. Henriksen, K. Andresen, F. Helgstrand, R. Aldemyati, J. Rosenberg

**Affiliations:** ^1^ Department of Surgery, Faculty of Medicine, University of Tabuk, Tabuk, Saudi Arabia; ^2^ Department of Surgery, Center for Surgical Science, Zealand University Hospital, Køge, Denmark; ^3^ Digestive Disease Center, Bispebjerg Hospital, University of Copenhagen, Copenhagen, Denmark; ^4^ Department of Surgery, Herlev Hospital, University of Copenhagen, Copenhagen, Denmark; ^5^ Department of Surgery, Faculty of Medicine, King Abdulaziz University, Rabigh, Saudi Arabia

**Keywords:** parastomal hernia, conservative management, abdominal binder, stoma care, quality of life

## Abstract

**Purpose:**

Parastomal hernia (PSH) is a frequent and challenging complication following stoma formation. While operative repair remains an important option in selected cases, non-operative strategies are essential, particularly for asymptomatic patients or those with significant comorbidities. This narrative review aims to synthesize current evidence on conservative management of PSH and on selected perioperative supportive measures, outlining core components, limitations, and research priorities.

**Methods:**

A narrative review of the literature was conducted focusing on non-operative strategies in PSH, including watchful waiting, core training, abdominal support garments, stoma care, and psychosocial considerations. Relevant publications were identified through searches in PubMed and Google Scholar (2011–2025) and supplemented by expert consultation.

**Results:**

In patients with minimal symptoms, conservative approach is widely accepted, given that recurrence rates have been reported to be comparable between elective and emergency repairs. Physical activity and patient education are underexplored yet promising components in enhancing function and improving quality of life. The role of abdominal binders remains empirically supported but lacks high-quality, PSH-specific evidence. Stoma care optimization - and psychosocial support are critical to improving quality of life but remain underrepresented in clinical guidelines. Most available data are extrapolated from ventral and incisional hernia literature, underscoring the need for targeted PSH research.

**Conclusion:**

Non-operative management remains a cornerstone of PSH care, requiring an individualized, multidisciplinary approach. Non-operative strategies should emphasize structured follow-up, abdominal support, guided core training, appliance adaptation, and psychosocial wellbeing. Dedicated prospective studies are urgently needed to define evidence-based protocols specific to PSH.

## Introduction

Parastomal hernias (PSH) are among the most challenging types of hernias to manage, both surgically and non-operatively. They commonly occur after stoma creation, with incidence rates reported as high as 50% within 2 years [1]. Even if a PSH is surgically repaired, approximately one in three patients will require reoperation for recurrence within 6 years [2].

Although surgical strategies have evolved significantly, the risk of recurrence is high. Therefore, non-surgical strategies—such as watchful waiting, stoma care, guided core training, and abdominal binders—are widely used to improve comfort and potentially slow progression, yet consensus remains lacking. Furthermore, comprehensive care should address psychological and social impacts of living with a PSH.

Stoma care and appliance management are central to the daily experiences of individuals with PSH. Herniation can distort the peristomal skin contour, complicating appliance fitting and increasing the risk of leakage, skin irritation, and dermatitis. Ripoche et al. reported that 28% of patients experienced leakage due to appliance-fitting issues, highlighting the importance of effective stoma management, as these complications can significantly impair quality of life and lead to social withdrawal [3].

This narrative review summarizes current non-operative approaches for the holistic management of PSH, emphasizing physical activity, abdominal support garments, and patient-centered care. It explores the role of guided core training, stoma care strategies, and patient education in enhancing quality of life. In addition to conservative strategies, the review also considers selected perioperative supportive measures—particularly the use of abdominal binders—as these overlap conceptually and remain clinically relevant. Furthermore, the review addresses psychosocial implications and underscores the importance of individualized, multidisciplinary management based on available evidence and clinical expertise.

## Methods

This work was designed as a narrative review of the literature on non-operative strategies for the management of parastomal hernia (PSH). We performed electronic searches in PubMed and Google Scholar covering the period from 2011 to March 2025. The following keywords and combinations were used: “parastomal hernia,” “conservative management,” “non-operative treatment,” “support garment,” “abdominal binder,” “stoma care,” “core training,” “physical activity,” and “quality of life.”

Studies were limited to the English language. Reference lists of included articles were manually screened for additional relevant publications. In addition, domain experts were consulted to identify key publications that may not have been captured through database searches. Because this is a narrative rather than a systematic review, no formal quality assessment or meta-analysis was performed; instead, emphasis was placed on summarizing available evidence and identifying knowledge gaps.

### Watchful Waiting

For patients with minimal symptoms, watchful waiting remains the common practice. Current evidence indicates that most PSH do not progress rapidly to severe complications, such as bowel obstruction or strangulation, allowing many patients to maintain good quality of life without surgical intervention for extended periods [1, 4].

Nevertheless, surgical outcomes highlight the trade-offs between elective and emergency repair. In a large cohort study of older adults, elective PSH repair was associated with a 40.2% complication rate, a 21.1% 5-year reoperation rate [5], and up to 35% recurrence rate [6].

However, emergency presentations, although infrequent, are associated with substantial complication rates of approximately 62% [7], and recurrence following acute repairs up to 38% [8].

### Physical Activity

Evidence regarding physical activity in patients with a stoma remains limited and generally of low quality. Most studies focus on cancer survivors and emphasize basic functional outcomes, with scarce data on optimal exercise regimens or broader quality of life parameters [9].

Several stoma-related factors may restrict physical activity. For example, stoma prolapse often compromises appliance adhesion, while skin discomfort further discourages mobility and exercise [10]. Despite these barriers, maintaining physical activity is of course important.

Among patients who are not surgical candidates or choose conservative management, fear of worsening the hernia or causing complications often leads to a sedentary lifestyle. This fear is well-documented and associated with physical deconditioning and social withdrawal [11, 12]. However, emerging evidence suggests that light to moderate physical activity is not only safe but beneficial in preserving general health and quality of life [13].

In patients undergoing surgical repair—whether through local fascial approximation, mesh reinforcement, or stoma relocation—postoperative activity guidance is essential to support healing, reduce recurrence risk, and restore functional independence. Unlike groin or small ventral hernias, the abdominal wall surrounding the stoma is structurally compromised and subject to complex, multidirectional stressors that challenge rehabilitation and long-term recovery [14].

Patients are typically advised to avoid heavy lifting, core straining, or high-impact movements for 2–4 weeks, with a gradual return to full activity based on individual healing and clinical judgment [15].

Targeted education about safe lifting techniques, optimal posture, and core-friendly exercises significantly reduces patient anxiety and enhances recovery outcomes, proving cost-effective in practice [16, 17]. Multidisciplinary involvement, particularly the inclusion of physiotherapists and stoma care nurses, is beneficial, especially for patients with complex hernias, reduced baseline fitness, or psychological distress [18].

### Abdominal Binders and Support Garments

The use of abdominal binders or support garments is more prevalent and clinically significant in patients with PSH than in those with other types of hernias [19]. These non-operative devices are relevant in two distinct contexts: conservative management for patients who are not surgical candidates, and perioperative supportive care following hernia repair.

#### Conservative Use

Support garments are commonly used as a conservative approach for symptom relief in patients managed without surgery. They are designed to provide targeted external compression around the stoma and hernia site, aiming to reduce protrusion, improve comfort, and enhance a sense of security during physical activity. Garments vary in form and compression level, with multiple types reported in the literature [19–21].

A recent scoping review found that binders are the most commonly recommended conservative treatment for PSH [21]. However, it is well-documented that many of them remain unused [19, 20, 22]. In a survey of 322 patients who had ordered a hernia belt, only 45% used it regularly, and just 27% believed it was the best way to manage their hernia [23]. Choosing the appropriate belt requires individualized assessment of patient expectations, symptoms, needs, and comorbidities [19, 20]. Practical testing of different belt types, access to tailored information, and follow-up were identified as key enablers of belt adherence in a qualitative Danish study [19].

Belt–appliance compatibility is crucial to avoid pressure ulcers, and in irreducible hernias, use should be cautious due to strangulation risk [21]. Well-fitted belts may relieve pain, bulging, leakage, and functional limitations, supporting activity and social participation. On the other hand, adverse effects include discomfort, restricted mobility, skin irritation, and ulcers [23–25].

Proper application technique is important: belts should ideally be applied while lying down with the hernia reduced, although this may be challenging for some patients [19, 21]. Given the complexity of selection and fitting, surgeons are encouraged to refer or re-refer patients with a PSH to a stoma care nurse for detailed assessment and patient education [19, 20].

#### Perioperative Supportive Care

In the postoperative phase, abdominal binders or support garments are often employed to protect the surgical repair site during the early healing, although this practice is supported mainly by evidence from incisional and ventral hernia repair literature, with limited data available for PSH. Several studies have suggested potential benefits of binder use, including reduced postoperative psychological distress [26], a lower incidence of surgical site infection [27], alleviation of pain [28–31] and enhanced mobilization [29–31].

However, evidence remains mixed. A patient survey following incisional hernia repair reported that although abdominal binders were frequently associated with reduced pain, approximately one-third of patients experienced decreased mobility. Notably, neither binder use nor prolonged physical rest appeared to significantly affect postoperative morbidity [32].

From randomized data of patients undergoing laparoscopic umbilical or epigastric hernia repair, binder use did not significantly influence postoperative pain or seroma formation, despite 86% of patients reporting subjective benefits [33]. A recent multicenter pilot trial involving laparoscopic IPOM for incisional hernia demonstrated a statistically significant reduction in early postoperative pain with binder use, although outcomes related to seroma formation and mobility remained unaffected [34].

Thus, although binders may not decrease risk of surgical complications there is indications that they improve patients’ satisfaction which is considered a core outcome in non-malignant surgery, including ventral hernia repair [22]. In clinical practice, therefore many healthcare providers recommend that patients wear support garments during upright activities for an extended period postoperatively—typically between 6 and 12 weeks, though some may continue for up to one year—due to the absence of standardized duration guidelines [35, 36].

#### Education and Fitting

Education on garment use is essential in both the conservative and perioperative settings. Patients should ideally be assessed and fitted by a trained stoma care nurse or clinical specialist, in line with the recommendations of the Canadian best practice (2024) and recent rapid review [37]. Instruction should address garment selection, correct fitting, timing and duration of wear, early recognition of issues such as excessive pressure or discomfort, and practical integration into daily activities including work, exercise, and social participation [17, 38, 39].

### Psychosocial Impact and Quality of Life

PSH present a distinct array of challenges that extend beyond the visible protrusion at the stoma site. These include stoma-related complications, cosmetic and negative body image issues, psychological distress, and functional limitations [19]—all of which require individualized, multidisciplinary care.

The psychosocial impact of PSH is profound yet often under-recognized. Patients frequently report anxiety, shame, and concerns about body image related to visible bulging or the potential for appliance leakage [40]. In one study using the Body Image Questionnaire, patients with PSH expressed significantly greater levels of distress and embarrassment [41]. These issues are particularly prominent among younger or working-age individuals and those returning to physically demanding roles. Reports suggest that up to 35% of individuals with PSH experience social limitations as a result of their condition [42].

Quality of life assessments and symptom scores—such as the Colostomy Impact Score or Hernia-Related Quality of Life (HerQLes) questionnaire—can serve as helpful tools to identify patients whose symptoms interfere with daily life [43], even in the absence of overt complications. In addition, surgical outcomes are not a convincing measure on their own; treatment of ostomy patients should be directed towards improving quality of life [44].

## Discussion

Despite advancements in surgical techniques, non-operative management remains essential, particularly for patients with minimal to moderate symptoms or significant comorbidities [4]. The current narrative review highlights that a holistic approach—integrating watchful waiting, physical activity, abdominal support garments, and multidisciplinary care—constitutes a cornerstone of effective PSH management and a doable alternative to surgery (Table 1). However, even in patients undergoing surgical repair, many of these approaches can most probably be of benefit both before and after operation.

**TABLE 1:** Practical Recommendations of the non-operative consideration regarding Parastomal Hernia.

Domain	Practical recommendations
Watchful waiting	Conservative approach is a widely accepted strategy as surgical repair is associated with high recurrence and complication rates
Physical Activity	Encourage guided core training and gradual return to activity to preserve abdominal wall function
Abdominal binders	Use in symptomatic patients for comfort and mobility; exercise caution in non-reducible PSH, ensure specialized stoma nurse assessment, check appliance compatibility, and provide education on correct use
Stoma care and appliance management	Ensure individualized fitting and education by a specialized stoma nurse, with appliance modifications to prevent leakage and skin complications
psychosocial impact	Offer counseling to address emotional distress and utilize validated QoL assessment tools
Multidisciplinary care	Foster collaboration among surgeons, physiotherapists and specialized stoma nurses

PSH, parastomal hernia, QoL: quality of life.

Watchful waiting is widely accepted as a reasonable initial strategy for patients with minimal or no symptoms, based on current evidence suggesting slow progression and minimal immediate risk of severe complications. This is also emphasized by the high recurrence rates after surgical repair, typically in the range of 21%–40% [2, 5–8].

Physical activity constitutes an essential yet under-investigated aspect of non-operative PSH management. Despite limited direct evidence for PSH, cautious extrapolation from ventral and incisional hernia literature suggests that targeted physical activity—guided by safety principles and gradual progression—can support recovery and reduce recurrence risk. Patient counseling should emphasize the high recurrence rates post-surgical intervention, reinforcing long-term strategies for core protection and activity modification [1]. Where available, physiotherapists and stoma care nurses collaborate in providing tailored rehabilitation programs, guiding patients through early-stage recovery, gentle strength-building, and confidence-boosting mobility routines. Nonetheless, robust evidence remains scarce, underscoring a clear need for more dedicated research into structured physiotherapy and rehabilitation protocols tailored explicitly to PSH.

The use of abdominal binders or support garments presents another critical aspect of non-operative PSH management, relevant both conservatively and perioperatively. Conservative use aims primarily at symptom relief and improved comfort during daily and physical activity, yet selection and adherence challenges persist. There is currently no evidence that wearing a belt affects the progression of a parastomal bulge over time, nor regarding optimal wear duration, compression level, or whether the belt should have a hole for the stoma [21]. Caution is particularly advised for non-reducible hernias due to pressure-related complications.

Although tapering strategies have not been formally studied, the limited duration of demonstrated benefits—such as reduced pain and reduced infection risk early after repair—suggests that gradual tapering of binder use may be reasonable as healing progresses, helping to minimise dependency. This approach is particularly important given individual variations in stoma location, mesh placement, and postoperative abdominal contour.

Beyond physical support, effective appliance management and stoma care, facilitated by skilled stoma nurses, significantly influence daily experiences and quality of life for individuals with PSH. Appliance modifications—including convex wafers, barrier rings, or custom-fitted pouches—are essential to mitigate complications such as leakage, skin irritation, and dermatitis, which significantly impair quality of life [3, 20].

Although the psychosocial impact of PSH is increasingly acknowledged, it remains inadequately addressed in clinical guidelines, which predominantly focus on surgical outcomes [41]. Patients frequently experience anxiety, embarrassment, and social withdrawal due to visible bulging and leakage fears. Proactive, empathetic clinical consultations addressing emotional responses, offering psychological support, and utilizing validated quality of life assessments are important. These tools not only aid in clinical decision-making but also help track non-operative management effectiveness.

Finally, elective repair should be carefully considered in light of recurrence rates that are comparable to emergency repairs [5–7]. Active watchful waiting, incorporating patient education, lifestyle measures, and regular follow-up, can help avoid urgent interventions while optimizing overall patient wellbeing.

## Limitations

It is noteworthy that most available evidence on non-operative strategies stems from incisional hernia repairs, which differ anatomically and biomechanically from PSH. As such, direct extrapolation should be approached with caution until PSH-specific trials are conducted. Management decisions must account for heterogeneity in hernia morphology and patient presentation. The European Hernia Society classification, which stratifies PSH by defect size (using 5 cm as a threshold) and the presence of a concomitant incisional hernia, offers a clinically relevant framework for individualized assessment [44]. While overlapping features between large PSH and incisional hernias may justify applying insights from ventral hernia literature in selected cases, further clinical research tailored to PSH is essential.

## Conclusion

Non-operative strategies for PSH management represent a critical component of personalized care and offer meaningful symptom relief, promote patient autonomy, and support quality of life, particularly for individuals not suited for immediate surgical intervention. However, standardized protocols and high-quality, PSH-specific research are clearly needed. Until then a holistic, multidisciplinary approach and collaboration is considered vital for optimal outcomes.
